# Effect of Hydrogen Oxide-Induced Oxidative Stress on Bone Formation in the Early Embryonic Development Stage of Chicken

**DOI:** 10.3390/biom13010154

**Published:** 2023-01-12

**Authors:** Yuguo Tompkins, Guanchen Liu, Brett Marshall, Milan Kumar Sharma, Woo Kyun Kim

**Affiliations:** Department of Poultry Science, University of Georgia, Athens, GA 30602, USA

**Keywords:** chicken embryo, bone formation, oxidative stress, ROS, H_2_O_2_

## Abstract

The current study aimed to monitor the impact of H_2_O_2_-induced oxidative stress on avian bone formation during the early stage of embryonic development. Fertilized Cobb broiler eggs were divided into five treatment groups and micro-injected with varying concentrations of H_2_O_2_, i.e., control (PBS; 0 nM), 10 nM, 30 nM, 100 nM, and 300 nM, on embryonic day 3, with continued incubation thereafter. The treatment concentrations were selected based on the level of lipid peroxidation and the survival rate of embryo. Embryos were collected at 6 h, 24 h, 48 h, and 72 h post-injection. The mRNA expression levels of apoptotic markers, antioxidant enzymes, and early bone formation gene markers were measured. The results showed that the microinjection of H_2_O_2_ altered the expression pattern of antioxidant enzymes’ mRNA during early embryogenesis and decreased the expression of *COL1A2* and *COL2A1* at 6 h and 24 h post-injection. Decreased expression of *BMP, BGLAP*, and *RUNX2* was observed 48 h post-injection. Additionally, a shorter embryo length was observed in the 100 nM and 300 nM H_2_O_2_ treatment groups 72 h post-injection. In conclusion, H_2_O_2_-induced oxidative stress suppressed the expression of bone formation gene markers, with chronic effects on avian embryonic development.

## 1. Introduction

The intensive system of poultry production places broilers under many stressors, resulting in exposure to oxidative stress. This is typically defined as an excess of reactive oxygen species (ROS) in relation to the ability of a biological system to neutralize them [1,2]. A relatively low level of ROS is essential to initiating and maintaining biological functions. However, excess ROS or long-term ROS exposure is harmful, leads to DNA damage, protein denaturation, and lipid peroxidation in both mammalian and avian species [3,4].

The role of oxidative stress in fetal growth and fetal programming via epigenetic mechanisms is well-discussed in mammals and humans [4,5,6]. In mammals, fetal growth is sensitive to oxidative stress because of a low antioxidant capacity [4]. Fetal oxidative stress can compromise fetal growth and impairs fetal skeletal formation [7]. Oxidative stress has been widely accepted as a mediator of pathogenesis in human bone diseases, with an increased level of ROS in osteoblasts being a hallmark element in the pathophysiology of bone loss [8]. Meanwhile, there has been a growing interest in oxidative stress in chicken production in recent years. The avian embryo develops in a closed system using the nutrients available within the egg before hatching. However, excessive ROS generated in tissues can deplete the antioxidant supply and cause avian embryonic oxidative stress [9]. Oxidative stress has been associated with early embryonic death, malformations, and post-hatch growth retardation in avian and mammalian species [10,11]. Conversely, maternal dietary supplementation of antioxidants may partially alleviate the deleterious impact of oxidative stress in chicks [12,13]. Egg quality and management are essential for limiting oxidative stress in the embryo, because stress factors such as low maternal diet quality or dietary exposure to contamination [14] and poor husbandry, including prolonged egg storage time or temperature stress, can significantly reduce hatchability and chick quality by impairing the antioxidant capacity [10,12,15,16,17].

Due to artificial selection, rapid muscle accretion at the expense of skeletal health has appeared in broiler chickens over the past few decades [18,19]. A number of studies have investigated various sources of oxidative stress and the oxidative machinery during the late stage of embryonic development in chickens [20]. Several methods have been used to induce avian embryonic oxidative stress, such as maternal manipulation and amniotic sac injection [21,22,23,24]. However, knowledge of the effects of oxidative stress on avian early embryonic bone development is limited. Evidently, in murine studies, bone stems cells’ proliferation and differentiation in the stem niche are often pre-programmed at early stages and have a significant impact on the future skeletal homeostasis [25]. Thus, the current study aimed to present direct evidence of the relationship between embryonic ROS and developmental retardation in the developing chicken embryo. The expression patterns of several sets of genes in osteogenesis, chondrogenesis, and apoptosis, as well as antioxidant enzyme expression from embryonic day 3 (ED 3) to embryonic day 7 (ED 7) were assessed.

## 2. Materials and Methods

### 2.1. Ethics Statement

All experiments followed the guidelines of the Institutional Animal Care and Use Committee and were conducted at research center of the Department of Poultry Science, University of Georgia, Athens, GA, USA.

### 2.2. H_2_O_2_ Microinjection

**Figure 1:**
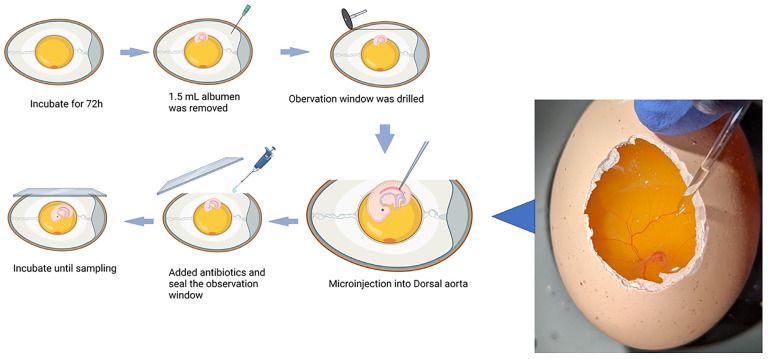
Illustration of the microinjection of chicken embryos at ED 3. The dorsal aorta was pierced using the tip of a micropipette, and the same volume of a hydrogen peroxide (H_2_O_2_) solution at different concentrations was injected into Cobb500 embryos. Glass coverslips (24 × 55 mm) were used to seal the observation windows, and the treated embryos were returned to the incubator without turning. Illustration created with BioRender.com.

The injection protocol was adapted from previous publications [26,27]. Cobb500 fertilized eggs (Cleveland, GA, USA) were set on their long axis in a bench incubator (GQF 1502, Savannah, GA, USA) without egg turning, at 38 °C and 50% relative humidity. At 72 h of incubation (Embryonic day 3; ED 3), the eggs were sanitized with 70% ethanol, and 1.5 mL of the albumen was removed with an 18-gauge needle after piercing the prolate end, followed by sealing with hot glue to avoid contamination or dehydration. An observation window was carefully drilled on the eggshell at the apex of the long axis, and the eggs were placed under a stereomicroscope for microinjection (Olympus, Center Valley, PA, USA). H_2_O_2_ was diluted to the proper concentrations, and heat-pulled capillary micropipettes were loaded with H_2_O_2_ or PBS and mounted onto an aspirator. The range of the treatment concentration of H_2_O_2_ was selected based on our previous in vitro study, and the embryo mortality rate was evaluated. The dorsal aorta was pierced using the tip of the micropipettes (illustrate in Figure 1). The same volume of treatment solutions was injected into each sample (10 pumps for each sample); the final concentrations of H_2_O_2_ (30% (*w*/*w*) solution Sigma-Aldrich, St. Louis, MO, USA) were 0 nM (PBS), 10 nM, 30 nM, 100 nM, and 300 nM. Following the injections, 100 µL of 1× antibiotics (antibiotic antimycotic solution, 100× stock, containing 10,000 units of penicillin, 10 mg of streptomycin, and 25 µg of amphotericin B /mL; Sigma-Aldrich) was added. Glass coverslips (24 × 55 mm; ThermoFisher Scientific, Waltham, MA, USA) were used to seal the observation windows, and the treated embryos were returned to the incubator without turning. A total of 320 eggs were injected (16 eggs × 5 treatments × 4 time points), and 8 embryos with regular and non-significantly altered heartbeat rates were kept for tissue collection.

### 2.3. Embryonic Survival Rate and Embryo Length Measurements

The embryos’ heartbeat and the embryonic survival rate after microinjection were monitored through the glass coverslips, and both data were used to determine the toxicity level of the H_2_O_2_ concentrations applied in the present study. The number of dead embryos was recorded and these embryos were removed. The embryos with regular and non-significantly altered heartbeat rates were kept for tissue collection. The length of the embryos was measured with a digital caliper (H-7352, ULINE, Kenosha, WI, USA) on ED7. For mRNA expression, eight live embryos from each treatment groups were collected at each time point. The embryos were sacrificed by transverse cut 6 h and 24 h post-injection and by decapitation 48 h and 72 h post-injection. Whole embryos were harvested 6 h, 24 h, 48 h, and 72 h post-injection and stored at −80 °C until the analysis.

### 2.4. Lipid Peroxidation and Antioxidant Status Assay

To determine the toxicity level of each H_2_O_2_ concentration, embryonic tissue lipid peroxidation was determined by using the QuantiChrom TBARS Assay Kit (BioAssay Systems, Hayward, CA, USA). Embryo tissue samples were collected 72 h post-injection, snap-frozen in liquid nitrogen, and then kept at −80 °C. Then, 30 mg of sample was homogenized and centrifuged in the assay buffer, and all assay procedures were performed according to the manufacturer’s protocols. Protein concentration was measured by a protein quantification assay (Pierce™ BCA Protein Assay Kit, ThermoFisher Scientific) following the manufacturer’s instructions.

### 2.5. RNA Isolation, cDNA Synthesis, and Real-Time Polymerase Chain Reaction (qRT-PCR) Analysis

The embryo samples were homogenized, and 50 mg of the samples were used for RNA isolation. Embryonic total RNA was extracted by using the Qiazol reagent (Qiagen, Germantown, MD, USA) according to the manufacturer’s instructions. A Nano-Drop 1000 Spectrophotometer (ThermoFisher Scientific) was used to determine the quantity of the extracted RNA. The cDNA was synthesized from total RNA (2000 ng) using high-capacity cDNA reverse transcription kits (Applied Biosystems, Foster City, CA, USA). Real-time quantitative polymerase chain reaction (RT-qPCR) was used to measure the relative expression of specific transcripts. The primers were designed using the Primer-BLAST program (accessed on 1 September 2021; https://www.ncbi.nlm.nih.gov/tools/primer-blast/). The PCR products were size-verified by gel electrophoresis, and primer specificity was validated by melting curve analysis. The details of the primer sequences used for the experiment are presented in Table 1. RT-qPCR was performed on an Applied Biosystems StepOnePlus™ (ThermoFisher Scientific) apparatus with iTaq™ Universal SYBR Green Supermix (BioRad, Hercules, CA, USA) using the following conditions for all genes: 95 °C for 10 min followed by 40 cycles at 95 °C for 15 s, annealing temperature (Table 1) for 20 s, and extension at 72 °C for 1 min. The geometric mean of housekeeping genes including hydroxymethylbilane synthase (*HMBS*), glyceraldehyde-3-phosphate dehydrogenase (*GAPDH*), and actin beta (*ACTB*) was used for normalization. The stability of the housekeeping genes was confirmed by their consistent Ct values among the treatments (*p* > 0.1). The samples were run in duplicate, and the relative gene expression was analyzed using the 2^−ΔΔCt^. The mean ΔCt of each marker gene from the control group was used to calculate the ΔΔCt value, the 2^−ΔΔCt^ expression levels were normalized to 1 for the control group, and the expression levels in the other treatment groups are presented as fold change relative to the expression in the control group.

### 2.6. Statistical Analysis

All experimental data are expressed as mean with a standard deviation of the mean (SEM). The data were tested for homogeneity of variances. All gene expression data were analyzed by one-way ANOVA followed by Dunnett’s test. The embryonic survival rate means were submitted to two-way ANOVA by JMP Pro14 (SAS Institute, Cary, NC, USA), and the main effects and their interactions were considered. Statistical significance was set at *p* ≤ 0.05, and results with 0.05 ≤ *p* ≤ 0.1 are also presented to show the trend toward statistical significance [28,29]. Pairwise correlations (JMP Pro14) were evaluated for the expression of all antioxidant enzyme-coding genes and bone formation genes.

## 3. Results

**Figure 2:**
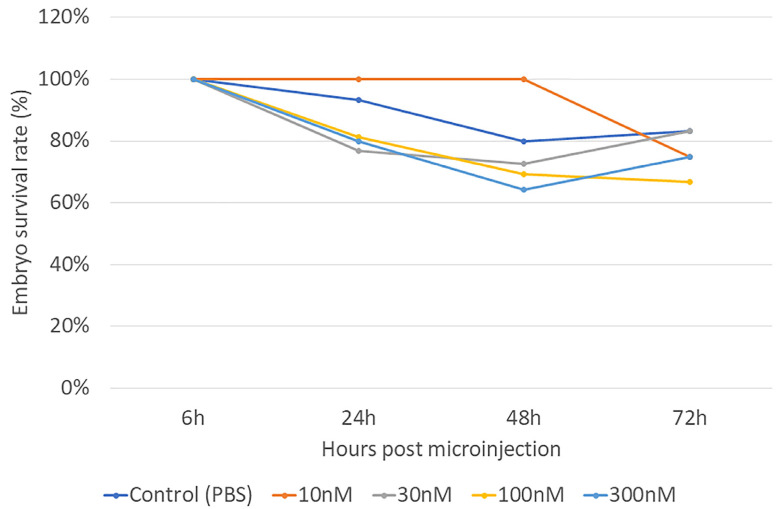
Embryo viability rate in the presence of different H_2_O_2_ concentrations 6 h, 24 h, 48 h, and 72 h post-injection. The embryo survival rate was used to determine the toxicity level of the injected concentrations of H_2_O_2_. The results showed that the mortality rate did not depend on the treatment dose (*p* = 0.226), but the incubation period had a significant impact on the survival rate (*p* = 0.003). The embryonic survival rate means were submitted to two-way ANOVA by JMP Pro14.

**Figure 3:**
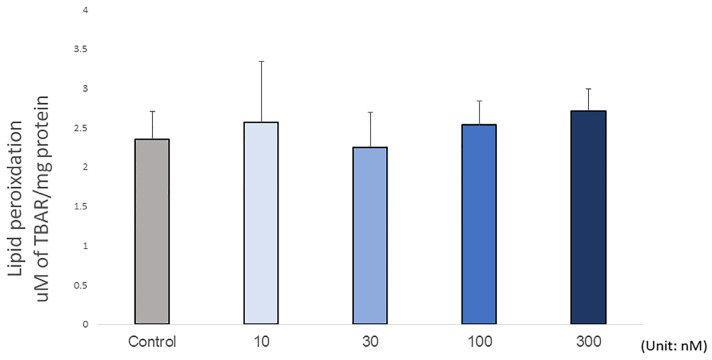
A lipid peroxidation assay was used to determine the toxicity level of the injected concentrations of H_2_O_2_. H_2_O_2_ did not alter the embryonic lipid peroxidation 72 h post-injection, indicating the treatment concentrations could be naturally converted to a non-embryonic toxic level by antioxidant enzyme degradation. The treatment groups were compared with the control group using the Dunnett’s test (*p* > 0.05; N = 8).

The embryonic survival rate after microinjection varied from 60% to 100% within 72 h post-injection (Figure 2). At 6 h post-injection, no embryonic mortality was observed in any group. At 24 h post-injection, the survival rate of the injected embryos decreased by around 10% compared to the PBS-injected controls. At 48 h post-injection, individuals injected with 300 nM H_2_O_2_ showed a 30% lower survival rate than those injected with 10 nM H_2_O_2_. At 72 h post-injection, the embryos injected with 100 nM H_2_O_2_ showed a lower survival rate than the PBS-injected controls. By comparing all the treatment groups at different time points, results showed that the mortality rate did not depend on the treatment dose (*p* = 0.226), but the incubation period had a significant impact on the survival rate (*p* = 0.003). There was no interaction between treatment dosage and incubation time (*p* = 0.878). Together, these data suggested that H_2_O_2_ did not significantly contribute to mortality. Supporting this, the lipid peroxidation results showing the level of malonaldehyde (MDA) in the embryo did not change with H_2_O_2_ treatment (*p* = 0.711; Figure 3), indicating that H_2_O_2_ did not cause embryonic tissue lipid peroxidation after 72 h post-injection. This result showed the doses of H_2_O_2_ were below the levels at which embryonic lipid peroxidation occurred 72 h post-injection. Together, these findings show that the concentrations of H_2_O_2_ used in the present study were safely below the levels at which any embryotoxicity occurs.

**Figure 4:**
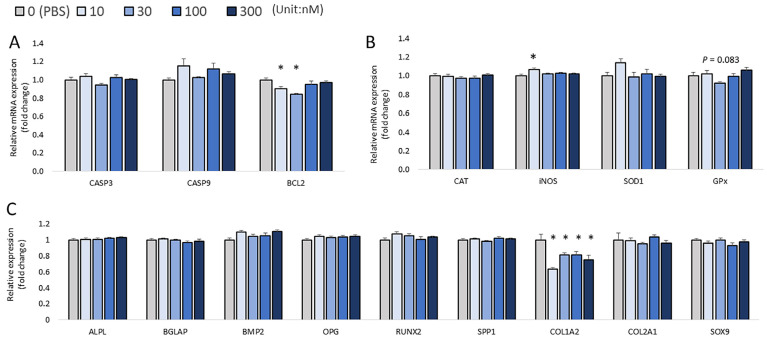
Effects of H_2_O_2_ on the mRNA expression of several sets of genes in chicken embryos at 6 h post-injection. (**A**) Apoptotic genes’ expression; (**B**) Antioxidant enzymes’ gene expression; (**C**) Bone formation biomarkers’ expression. Each value represents the mean ± SEM (N = 8). Treatments with * showed a significant difference compared with the control using the Dunnett’s test, *p* < 0.05.

At 6 h post-injection, 10 nM and 30 nM H_2_O_2_ significantly decreased the expression of *BCL2* compared with that in PBS-injected controls (*p* < 0.05; Figure 4A), and 10 nM H_2_O_2_ significantly increased the expression of *iNOS* compared with that in PBS-injected controls (*p* < 0.05; Figure 4B); H_2_O_2_ tended to alter the mRNA expression of *GPX1* among the treatments (*p* = 0.080; Figure 4B). In addition, a significantly decreased expression of *COL1A2* was observed in all H_2_O_2_ treatment groups compared with the control (*p* < 0.05; Figure 4C). For the correlation analysis, *BMP2* expression was positively correlated with the expression of *iNOS* (*p* < 0.001, R^2^ = 0.197). *BAGLAP* was positively correlated with the expression of *iNOS* (*p* < 0.001, R^2^ = 0.366).

**Figure 5:**
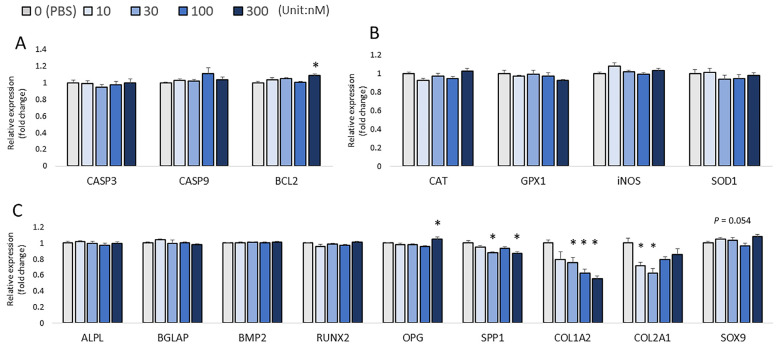
Effects of H_2_O_2_ on the mRNA expression of several sets of genes in chicken embryos at 24 h of post-injection. (**A**) Apoptotic genes’ expression; (**B**) Antioxidant enzymes’ gene expression; (**C**) Expression of bone formation biomarkers. Each value represents the mean ± SEM (N = 8). Treatments with * showed a significant difference compared with the control using the Dunnett’s test, *p* < 0.05.

At 24 h post-injection, the expression of *BCL2*, an anti-apoptosis regulator, was significantly upregulated by the 300 nM H_2_O_2_ injection when compared to the control (*p* < 0.01, Figure 5A). The results of the bone formation markers indicated a general decrease in the mRNA expression levels with the injection of higher concentrations of H_2_O_2_. Moreover, the 30 nM and 300 nM H_2_O_2_ concentrations significantly decreased the embryonic mRNA levels of *SPP1* (*p* < 0.05; Figure 5C). Furthermore, the 30 nM, 100 nM, and 300 nM groups had a lower level of *COL1A2* compared to the control (*p* < 0.05; Figure 5C). The expression of chondrocyte collagen *COL2A1* was inhibited by 10 nM and 30 nM H_2_O_2_ compared to the PBS-injected control (*p* < 0.05; Figure 5C). In contrast, injection of 300 nM H_2_O_2_ produced a significantly higher mRNA level of *OPG* when compared with the control (*p* < 0.05; Figure 5C), and the expression of *SOX9* showed an increasing trend for treatments with higher concentrations of H_2_O_2_ (*p* = 0.054; Figure 5C). However, the H_2_O_2_ treatment did not change the mRNA expression of *ALP*, *BGLAP*, *BMP2*, or *RUNX2* 24 h post-injection. For the correlation analysis, *RUNX2* expression was positively correlated with the expression of *SOD1* (*p* < 0.001, R^2^ = 0.253), and *BMP2* expression was positively correlated with the expression of *SOD1* (*p* = 0.002, R^2^ = 0.218) and *iNOS* (*p* = 0.002, R^2^ = 0.106).

**Figure 6:**
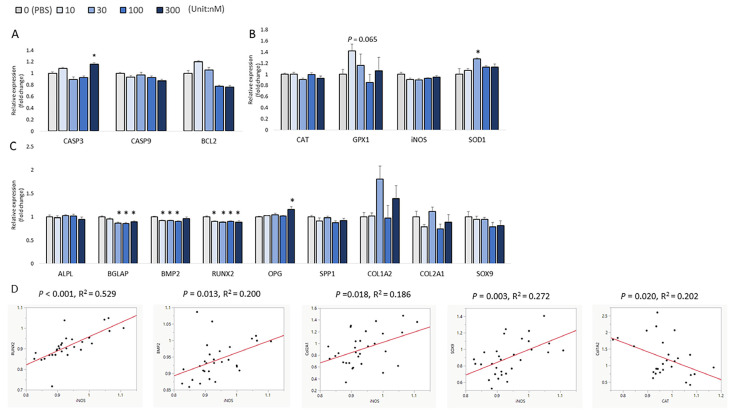
Effects of H_2_O_2_ on the mRNA expression of three sets of genes in chicken embryos at 48 h post-injection. (**A**) Apoptotic genes’ expression; (**B**) Antioxidant enzymes’ gene expression; (**C**) Expression of bone formation biomarkers. Each value represents the mean ± SEM (N = 8). Treatments with * showed a significant difference compared with the control using the Dunnett’s test, *p* < 0.05. (**D**) The correlation between antioxidant enzyme-coding genes’ expression and bone formation genes’ expression was evaluated by using pairwise correlations (JMP Pro14).

**Figure 7:**
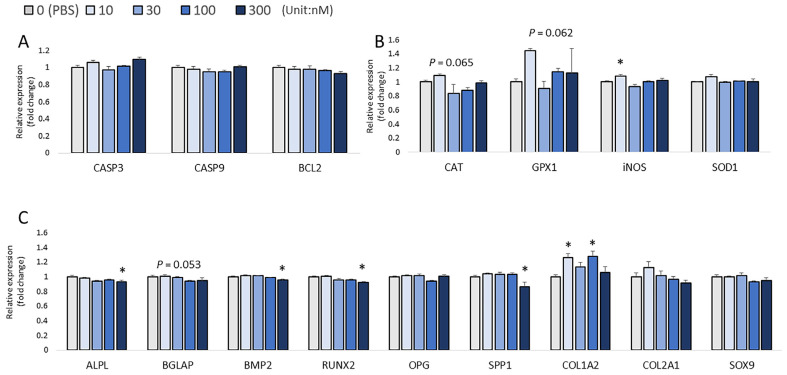
Effects of H_2_O_2_ on the mRNA expression of three sets of genes in chicken embryos at 72 h post-injection. (**A**) Apoptotic genes’ expression (**B**) Antioxidant enzymes’ gene expression (**C**) Expression of bone formation markers. Each value represents the mean ± SEM (N = 8). Treatments with * showed a significant difference compared with the control using the Dunnett’s test, *p* < 0.05.

At 48 h post-injection, 300 nM H_2_O_2_ caused a significant increase in the expression of *CASP3* compared to the control group (*p* < 0.05; Figure 6A). The 30 nM H_2_O_2_ treatment significantly increased the expression of *SOD1* compared to the control (*p* < 0.05; Figure 6B). An increasing trend in the expression of *GPX1* was observed in the low-H_2_O_2_ treatment groups (*p* = 0.065; Figure 6B). H_2_O_2_ has an inhibitory effect on embryonic bone formation. The expression of *RUNX2* was significantly decreased by 10 nM, 30 nM, 100 nM, and 300 nM H_2_O_2_ micro-injection as compared to the PBS-injected control group (*p* < 0.05; Figure 6C), whereas the expression of *BMP2* was significantly decreased in the groups injected with 10 nM, 30 nM, and 100nM H_2_O_2_ as compared to its expression in the control group (*p* < 0.05; Figure 7C). The mRNA expression of *OPG* was significantly increased by the 300 nM H_2_O_2_ injection compared to the expression in the PBS-injected controls (*p* < 0.05; Figure 6C). The mRNA expression of *BGLAP* was significantly decreased by 30 nM, 100 nM, and 300 nM H_2_O_2_ (*p* < 0.05; Figure 6C), whereas the H_2_O_2_ treatment did not change the mRNA expression of *SPP1*, *COL2A1*, *COL1A2*, and *SOX9* (*p* > 0.05). For the correlation analysis, *RUNX2* expression was positively correlated with the expression of *iNOS* (*p* < 0.001, R^2^ = 0.529; Figure 6D), and *BMP2* expression was positively correlated with the expression of *iNOS* (*p* = 0.013, R^2^ = 0.200; Figure 6D). *COL2A1* expression was positively correlated with the expression of *iNOS* (*p* = 0.018, R^2^ = 0.186; Figure 6D), and *SOX9* expression was positively correlated with *iNOS* expression (*p* = 0.003, R^2^ = 0.272; Figure 6D). However, *COL1A2* expression was negatively correlated with the expression of *CAT* (*p* = 0.020, R^2^ = 0.202; Figure 6D).

**Figure 8:**
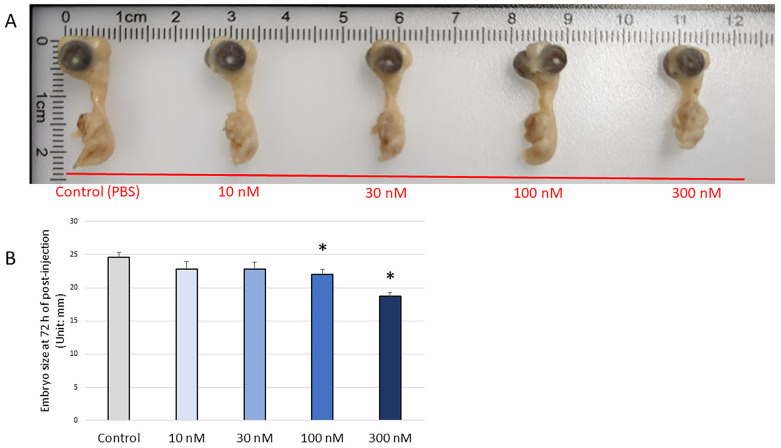
Embryos length is shorter for embryos microinjected with 100 nM and 300 nM H_2_O_2_ compared to that of the PBS-injected controls. (**A**) Embryo samples at ED 7 (72 h post-injection). (**B**) The length of the embryos was measured with a digital caliper. Each value represents the mean ± SEM (N = 8). Treatments with * showed a significant difference compared with the control using the Dunnett’s test, *p* < 0.05.

At 72 h post-injection, the lethality rates were up to nearly 35%, but the mortality rate did not depend on the treatment doses. In addition, there were no significant changes in the expression of apoptosis-related marker genes among the treatments (*CASP3*, *CASP9*, and *BCL2*; Figure 7A). The results showed an increasing trend in the expression of *CAT* (*p* = 0.065; Figure 7B) and *GPX1* (*p* = 0.062; Figure 7B) with H_2_O_2_ treatment. The injection of 10 nM H_2_O_2_ significantly increased the mRNA expression of *iNOS* (*p* < 0.05; Figure 7B). Additionally, the expression of *COL1A2* was found to be significantly increased after treatment with 10 nM and 100 nM H_2_O_2_ compared to the controls (*p* < 0.05; Figure 7C). The highest dose of H_2_O_2_ (300nM) suppressed the expression of *SPP1*, *ALP*, *RUNX2*, and *BMP2* compared to the control (*p* < 0.05; Figure 7C). Moreover, *BGLAP* expression showed a decreasing trend with H_2_O_2_ treatment (*p* = 0.053; Figure 7C). There was no difference in the expression of *OPG*, *COL2A1*, and *SOX9* (*p* > 0.05). For the correlation analysis, *RUNX2* expression was positively correlated with the expression of *iNOS* (*p* = 0.008, R^2^ = 0.233) and *GPX* (*p* = 0.023, R^2^ = 0.189). *COL2A1* expression was positively correlated with the expression of *SOD1* (*p* = 0.046, R^2^ = 0.140), and *SOX9* expression was positively correlated with *GPX1* expression (*p* = 0.003, R^2^ = 0.292). Moreover, a decreased embryo length was observed in the 100 nM and 300 nM H_2_O_2_ groups compared to the PBS-injected control (*p* < 0.05; Figure 8A,B).

## 4. Discussion

The current study showed that the microinjection of H_2_O_2_ altered the mRNA expression pattern of antioxidant enzymes during early embryogenesis (ED 3 to ED 7). While there are several potential delivery routes, such as the air cell, the amniotic sac, the yolk sac, or directly into the chicken embryos [21], the direct injection into circulatory system via the dorsal aorta ensures the delivery to the embryo and provides an immediate impact on the measurable gene expression [30,31]. In addition, the dorsal aorta is a morphogenetic signaling center that initiates a molecular cascade of bone formation and secretes bone morphogenetic proteins (BMPs) at an early development stage [32,33]. With the current injection method, H_2_O_2_-induced oxidative stress had a great chance of directly influencing the expression of key proteins for bone formation in an early embryonic development stage, and as the result showed, a suppressed collagen synthesis was observed under H_2_O_2_-induced oxidative stress. H_2_O_2_-induced oxidative stress suppressed bone formation gene markers’ expression with chronic effects. The pitfall of this injection method is that eggs have to be held without turning during the post-injection period, as turning the eggs after injection would not have been feasible in order to monitor embryo viability through the shell window. ED 3 to ED 7 represent a critical period for egg turning in artificial chicken egg incubation [34]. A lack of egg-turning is detrimental to hatchability and embryo growth. This possibly caused the low embryo viability rate at 72 h of the incubation study period.

H_2_O_2_ is an active oxidizing reagent that can react with proteins and lipids in embryos, and antioxidant defense systems elaborately protect against lipid peroxidation [13]. Previous studies have shown that the antioxidant content and antioxidant enzyme activity are positively correlated with embryonic growth rate [35,36,37]. In the present study, the expression levels of antioxidant enzymes’ genes were altered by low concentrations of H_2_O_2_. Specifically, there was an increase in *iNOS* expression by 10 nM H_2_O_2_ at 6 h and 72 h, as well as changes in the expression of *SOD1* at 48 h and a trend of changes in *GPX1* expression at 6 h, 48 h, and 72 h. These changes in the expression of antioxidant enzymes’ genes indicated the stimulation of the antioxidant defense system in the embryos. It has been noted that low nontoxic levels of oxidative stress are reversible by mediating the mRNA expression and activity of antioxidant enzymes [38,39]. Although the changed expression of the antioxidant enzymes’ genes was not dependent on the treatment doses in the present study, we hypothesized that the low and the high concentrations of H_2_O_2_ might interact with different pathways in regulating cellular metabolism, which either stimulated the antioxidant defense system or triggered cellular apoptosis.

The expression of several osteogenesis marker genes was used to assess the skeletal growth pattern under oxidative stress during early embryonic development in the present study. Although the chondrogenesis and osteogenesis are considered separate processes during endochondral bone formation, they closely share signaling pathways and can be considered as a continuous developmental process [40]. The avian embryonic skeletogenesis is modulated by signaling pathways including the Sonic hedgehog pathway, bone morphogenetic protein (BMPs) pathways, and fibroblast growth factor pathways [41,42]. Runt-related transcription factor 2 (RUNX2) is a downstream target of BMPs [41,43]. It is key a transcriptional hallmark to detect signaling changes in chondrification and osteogenesis during embryonic growth [27,44]. Moreover, regulated by the RUNX2 pathway, the expression of bone matrix protein genes, including bone gamma-carboxyglutamate protein (*BGLAP*), secreted phosphoprotein 1 (*SPP1,* osteopontin), collagen type I alpha 2 chain (*COL1A2*), SRY-box transcription factor 9 (*SOX9*), and collagen type II alpha 1 chain (*COL2A1*), are widely accepted biomarkers of new bone formation and growth [45]. With the current data, it was clearly observed that H_2_O_2_ has an inhibitory effect on bone formation after 72 h post-injection. More specifically, the mRNA of *COL2A1* was suppressed by H_2_O_2_ injection at 6 h post-injection. Decreased mRNA expression levels of *COL1A2* and *COL2A1* were observed 24 h post-injection. It is well known that collagen fibers are crucial in the primary mineralization process and bone strength [46]. The synthesis of collagen is regulated at the transcriptional and posttranslational levels [47]. Previous studies detected a drastically increased level of *COL1A1* and *COL2A1* RNA expression between ED 5 and ED 10 in chick embryos [48]. Meanwhile, inhibited production of collagen has been reported in various cell types after oxidative stress exposure, including muscle cells and cardiac fibroblasts [48,49,50]. Therefore, we hypothesized that collagen production could be interrupted by oxidative stress at an early stage of development, and the changed expression levels of bone-related collagens would stunt embryonic bone development at a later stage. In addition, an appropriate expression profile of the collagen types is essential to bone health, and changed expression patterns of collagen types have been associated with bone disorders in chickens. For example, chondrocytes derived from dyschondroplasic growth plates exhibited reduced type X collagen expression and increased type I collagen expression [51]. With the information above, it can be explained that the increased expression of type I collagen at 72 h post-injection possibly signified chondrocyte dedifferentiation. Oxidative stress-induced alteration in the expression ratio of collagen types in the early differentiation stage might produce more significant phenotype changes in future development.

It is well-documented the significance role of BMPs in skeletogenesis, which mediate chondrogenic differentiation and osteogenic differentiation [40,52,53]. RUNX2 functions as the master regulatory factor that controlling osteoblast progenitors’ proliferation, the commitment of stem cells to osteoblast lineage cells, and the expression of bone matrix protein genes [54]. RUNX2 also mediates chondrocyte hypertrophy and contributes to longitudinal bone growth [52,55,56]. Notably, there was a positive correlation between the expression of *iNOS* and that of *RUNX2* and *BMP2* 48 h post-injection (R^2^ = 0.529) in the present study. This result indicated a possible complex interplay between the presence of H_2_O_2_, NO production, and the inhibition of bone formation. The regulation of iNOS activity and NO production has been implicated in bone development and homeostasis [57,58]. NO production has been shown to have detrimental effects on chondrocyte function by inhibiting collagen synthesis and enhancing apoptosis [58]. However, the function of NO in stem cell differentiation capacity remains controversial. For example, studies using mouse bone cell lines have reported that cytokine-induced NO production inhibited osteoblast differentiation in vitro [57,59], while other studies demonstrated that NO stimulated bone marrow-derived mesenchymal stem cells (MSCs) to undergo osteogenic differentiation [60]. Therefore, more research is required to answer questions regarding the effect of NO production on chicken bone embryonic development.

Excessed ROS production results in oxidative damage, altering the cell fate decisions that can lead to structural and functional changes in developing animals during embryonic development [1]. Oxidative stress can be an important factor determining chick quality through its impact on bone growth at an early stage of development. In avian species, the closed egg limits the external resources of nourishment; thus, the natural egg content, such as yolk lipids and antioxidants, provides the antioxidant defense again oxidative stress [61,62]. Whether or not an increased oxidative challenge causes oxidative damage to a developing embryo is likely to depend on the capacity of yolk-derived antioxidants to protect the developing tissues [9]. The embryonic development process consumes a large portion of nature antioxidants. It has been reported that antioxidants such as vitamin E in newly hatched chicks were dramatically depleted during the first few days [30,63]. In addition, stress during hatching can waste antioxidants and result in antioxidant depletion in embryos, which ultimately causes chicken embryonic oxidative stress during chicken early development, causing a disadvantage to early post-hatch development. Thus, maternal antioxidant supplementation could benefit the offspring by improving their antioxidant defense and protecting the tissues of the progeny from oxidative injury [64]. We further propose that maternal antioxidant supplementation has the potential to protect bone homeostasis from potential oxidative stress damage and eventually contribute to bone structural and metabolic integrity during embryonic development.

## Figures and Tables

**Table 1:** Nucleotide sequences of the primers used for quantitative real-time RT-PCR.

Gene ^1^	Primer Sequence (5′-3′)	Product Length (bp)	Annealing Temperature (°C)	Accession
***GAPDH***	F-GCTAAGGCTGTGGGGAAAGT R-TCAGCAGCAGCCTTCACTAC	161	55	NM_204305.1
***HMBS***	F-GGCTGGGAGAATCGCATAGG R-TCCTGCAGGGCAGATACCAT	131	59	XM_004947916.3
***ACTB***	F-CAACACAGTGCTGTCTGGTGGTA R-ATCGTACTCCTGCTTGCTGATCC	205	61	NM_205518.1
***ALPL***	F-CGACCACTCACACGTCTTCA R-CGATCTTATAGCCAGGGCCG	140	60	NM_205360.1
***RUNX2***	F-ACTTTGACAATAACTGTCCT R-GACCCCTACTCTCATACTGG	192	60	XM_015285081.2
***BGLAP***	F-GGATGCTCGCAGTGCTAAAG R-CTCACACACCTCTCGTTGGG	142	57	NM_205387.3
***SPP1***	F-GCCCAACATCAGAGCGTAGA R-ACGGGTGACCTCGTTGTTTT	204	57	NM_204535.4
***BMP2***	F-TCAGCTCAGGCCGTTGTTAG R-GTCATTCCACCCCACGTCAT	163	57	XM_025148488.1
***OPG***	F-ACGCTTGTGCTCTTGGACAT R-CAGCGTAGTACTGGTCTGGG	193	60	NM_001033641.1
***COL1A2***	F-CTGGTGAAAGCGGTGCTGTT R-CACCAGTGTCACCTCTCAGAC	222	60	NM_001079714.2
***COL2A1***	F-GGACCAGCAAGACGAAAGAC R-TGTAGGCGATGCTGTTCTTG	189	59	NM_204426.2
***SOX9***	F-AGGAAGCTGGCTGACCAGTA R-CGTTCTTCACCGACTTCCTC	193	61	XM_046929245.1
***SOD1***	F-ATTACCGGCTTGTCTGATGG R-CCTCCCTTTGCAGTCACATT	173	58	NM_205064.1
***CAT***	F-ACTGCAAGGCGAAAGTGTTT R-GGCTATGGATGAAGGATGGA	222	60	NM_001031215.1
***iNOS***	F-CCTGTACTGAAGGTGGCTATTGG R-AGGCCTGTGAGAGTGTGCAA	66	58	NM_204961.2
***GPX1***	F-AACCAATTCGGGCACCAG R-CCGTTCACCTCGCACTTCTC	122	60	NM_001277853.2
***CASP3***	F-TGGTATTGAAGCAGACAGTGGA R-GGAGTAGTAGCCTGGAGCAGTAGA	103	60	XM_015276122.2
***CASP9***	F-ATTCCTTTCCAGGCTCCATC R-CACTCACCTTGTCCCTCCAG	130	60	XM_046931415.1
***BCL2***	F-GAGTTCGGCGGCGTGATGTG R-TTCAGGTACTCGGTCATCCAGGTG	92	63	XM_046910476.1

^1^*GAPDH*: glyceraldehyde-3-phosphate dehydrogenase; *HMBS*: hydroxymethylbilane synthase; *ACTB*: actin beta; *OPG*: TNFRSF11B, TNF receptor superfamily member 11b; *IL1B*: interleukin 1 beta; *SPP1*: secreted phosphoprotein, osteopontin; *BMP2*: bone morphogenetic protein 2; *BGLAP*: bone gamma-carboxyglutamic acid-containing protein (osteocalcin). *RUNX2*: runt-related transcription factor 2; *ALPL*: alkaline phosphatase, biomineralization associated; *COL1A2*: collagen type I alpha 2 chain; *COL2A1*: collagen type II alpha 1 chain; SOX9: SRY-box transcription factor 9; *CAT*: catalase; *SOD1*: superoxide dismutase; *GPX1*: glutathione peroxidase 1; *iNOS*: NOS2, nitric oxide synthase 2; *CASP3*: caspase 3, apoptosis-related cysteine protease; *CASP9*: caspase 9, apoptosis-related cysteine protease; *BCL2*: anti-apoptotic gene B-cell lymphoma 2.

## Data Availability

Data generated or analyzed during this study are included in this published article and available from the corresponding author upon reasonable request.
